# Supplementary material for: SpoIVA-SipL Complex Formation Is Essential for Clostridioides difficile Spore Assembly
Source: J Bacteriol. 2019 Mar 26;201(8):e00042-19. doi: 10.1128/JB.00042-19 (PMC6436350; doi:10.1128/JB.00042-19)
Supplement: Supplemental file 1 [file JB.00042-19-s0001.pdf]

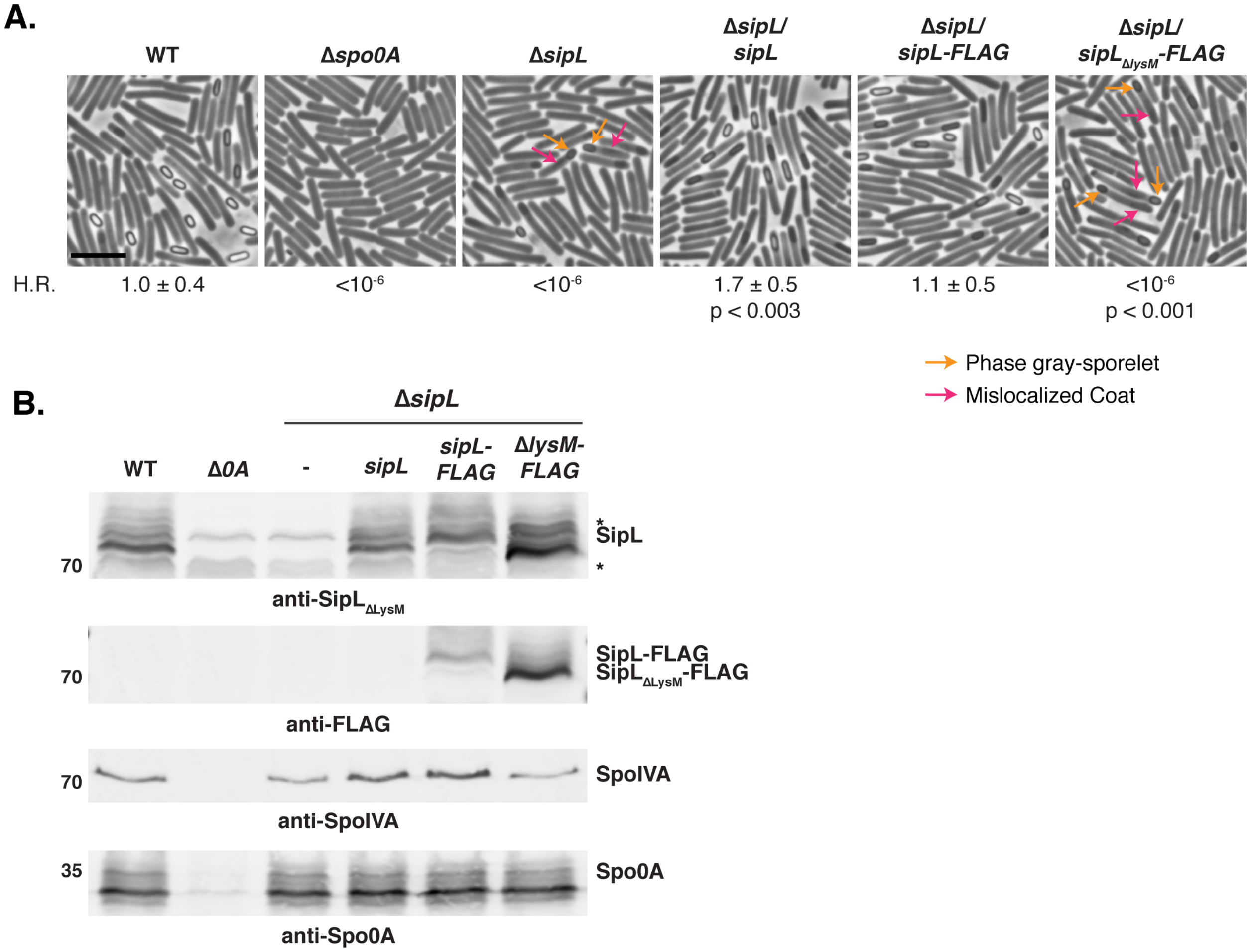

Figure S1. Complementation of Δ*sipL* with constructs encoding C-terminal FLAG tags. (A) Phase-contrast microscopy analyses of the Δ*sipL* strains complemented with either wild-type *sipL*, *sipL* encoding three FLAG epitopes at the C-terminus of SipL, or *sipL* encoding a LysM deletion along with three C-terminal FLAG epitopes. Cultures were analyzed ~20 hrs after sporulation induction. Arrows mark examples of sporulating cells with various defects in maturation: orange arrows highlight phase-gray sporelets (1), which appear to produce a cortex layer as evidenced by the phase-dark ring surrounding the forespore but do not become phase-bright, and pink arrows demarcate regions likely to be mislocalized coat (2, 3). Heat resistance efficiencies were determined from 20-24 hr sporulating cultures and represent the mean and

standard deviation for a given strain relative to wild type based on a minimum of three independent biological replicates. Statistical significance relative to wild type was determined using a one-way ANOVA and Tukey's test. (B) Western blot analyses of sporulating cultures shown in (A) using the indicated antibodies. Asterisks indicate non-specific bands detected by the anti-SipL$_{\Delta LysM}$ antibody. The addition of the three FLAG epitopes results in slower mobility of SipL-FLAG fusions relative to untagged SipL during SDS-PAGE.

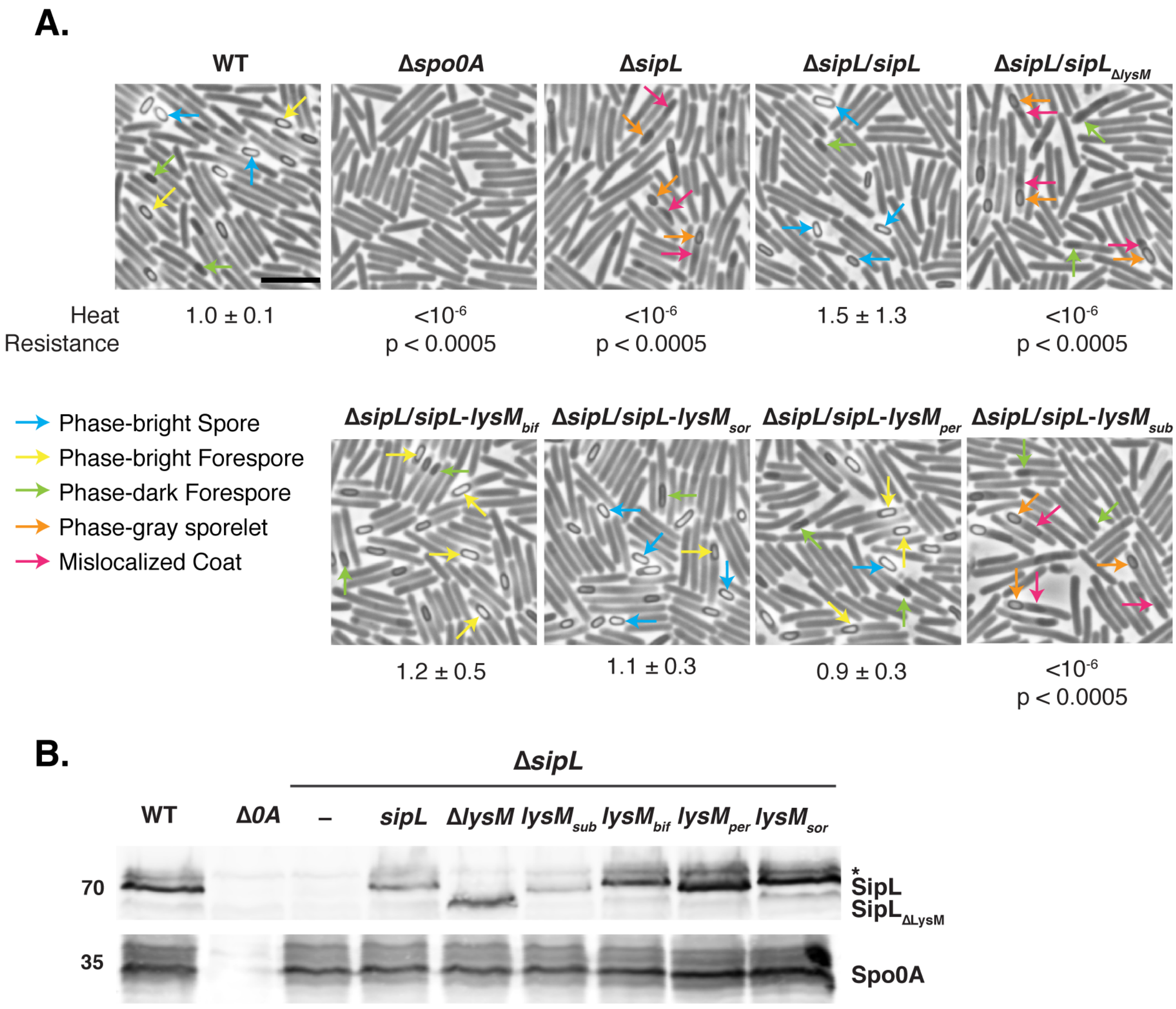

Figure S2. Complementation of Δ*sipL* with constructs encoding LysM domain swaps. (A) Phase-contrast microscopy analyses of the Δ*sipL* strains complemented with either wild-type *sipL* or *sipL* constructs in which the region encoding the LysM domain has been swapped with the corresponding region from clostridial SipL homologs or *B. subtilis* SpoVID. "*bif*" refers to *P. bifermentans*, "*sor*" refers to *P. sordellii*, "*perf*" refers to *C. perfringens*, and "*sub*" refers to *B. subtilis*. Cultures were analyzed ~20 hrs after sporulation induction. Arrows mark examples of sporulating cells with various defects in maturation: orange arrows highlight phase-gray sporelets (1), which appear to produce a cortex layer as evidenced by the phase-dark ring surrounding the forespore but do not become phase-bright; purple arrows highlight phase-bright

sporelets that appear swollen but are outlined by a phase-dark ring; and pink arrows demarcate regions likely to be mislocalized coat (2, 3). Heat resistance efficiencies were determined from 20-24 hr sporulating cultures and represent the mean and standard deviation for a given strain relative to wild type based on a minimum of three independent biological replicates. Statistical significance relative to wild type was determined using a one-way ANOVA and Tukey's test. (B) Western blot analyses of sporulating cultures shown in (A) using the indicated antibodies. Asterisks indicate non-specific bands detected by the anti-$SipL_{\Delta LysM}$ antibody.

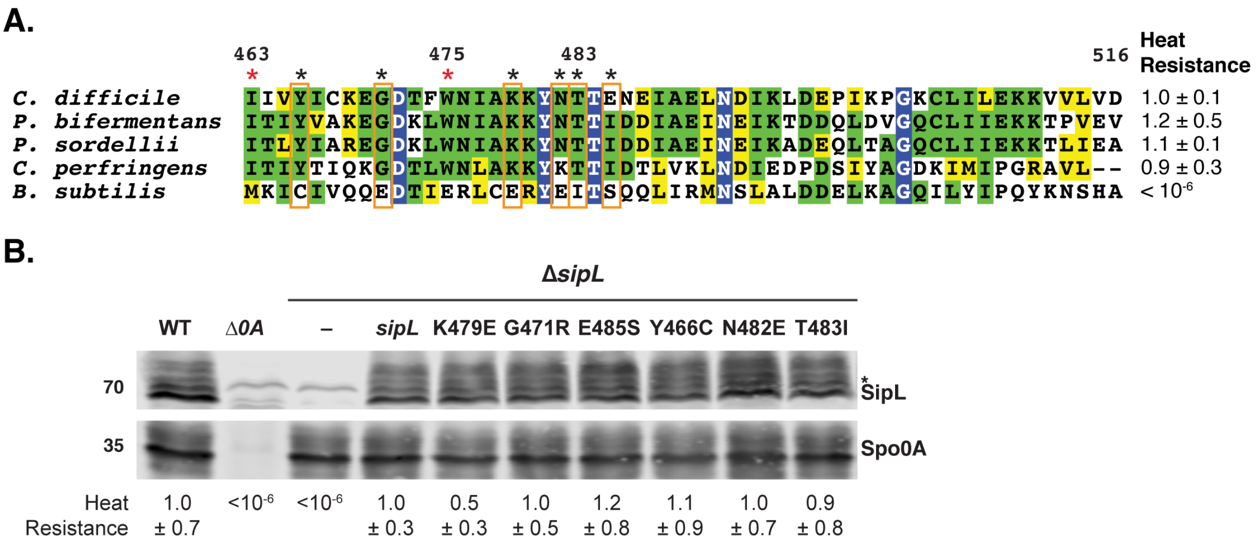

Figure S3. SipL point mutations that do not negatively impact SipL function. (A) Alignment of LysM domains from SipL homologs from *C. difficile* (CAJ70473), *P. bifermentans* (EQK49575)*, P. sordellii* (EPZ54296), and *C. perfringens* (YP_696893), as well as the LysM domain from *B. subtilis* SpoVID (NP_390689). Blue boxes with white text indicate residues that are completely conserved; green boxes indicate residues that are conserved in some of the homologs; and yellow boxes mark residues that are similar between the homologs. The red asterisks highlight residues whose mutations led to defects in functional spore formation (**Fig. 5**), while black asterisks indicate residues whose mutation to the residue in the *B. subtilis* SpoVID LysM domain did not result in significant defects in spore formation (shown here). The heat resistance of chimeric LysM strains relative to wild type is shown. The limit of detection of the assay is 10^{-6}. (B) Western blot analyses of sporulating cultures shown in (A) using the indicated antibodies. Asterisk indicates a non-specific bands detected by the anti-SipL$_{\Delta LysM}$ antibody. Heat resistance efficiencies were determined from 20-24 hr sporulating cultures and represent the mean and standard deviation for a given strain relative to wild type based on a minimum of three independent biological replicates.

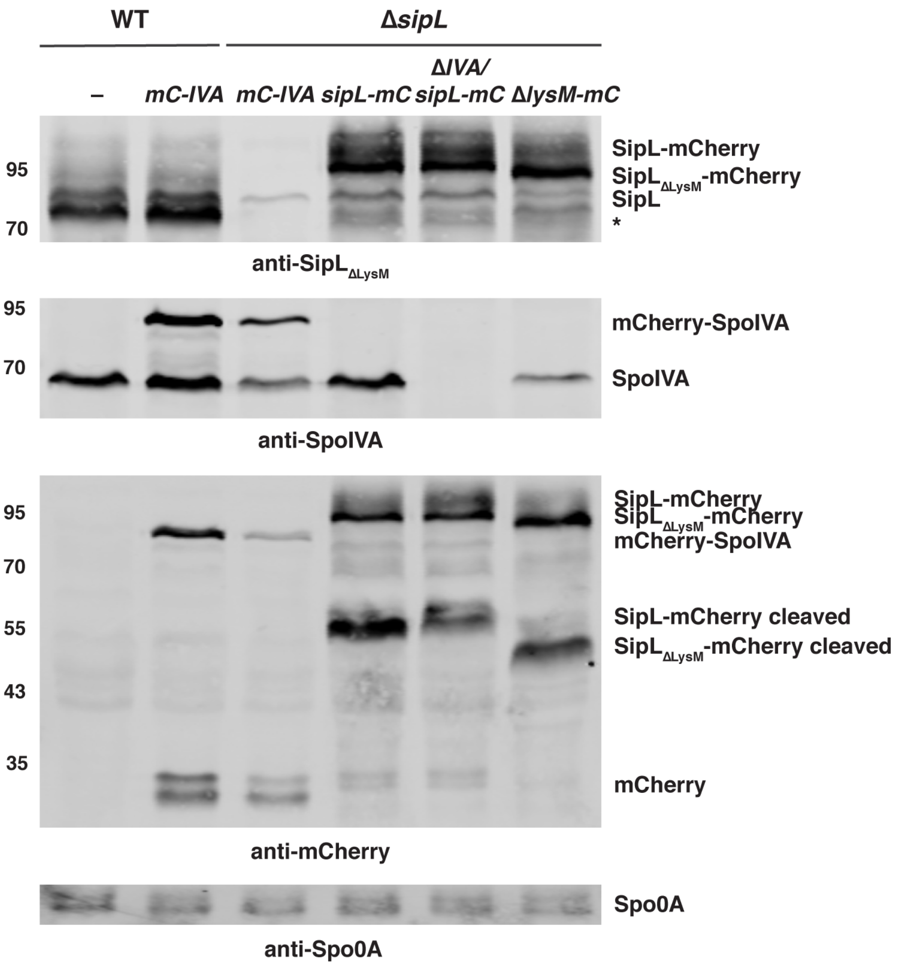

Figure S4. Western blot analyses of mCherry fusion proteins used in localization studies. Antibodies to mCherry (mC), SpoIVA, and SipL$_{\Delta LysM}$ were used as indicated. Asterisk indicates a non-specific band recognized by the SipL$_{\Delta LysM}$ antibody. Partial processing of mCherry-IVA releases free mCherry as previously reported (3). Several isoforms of SipL and SipL-mCherry are detected, most that run higher than expected. Processed forms of SipL-mCherry are marked. The western blots shown are representative of the results from two independent biological replicates.

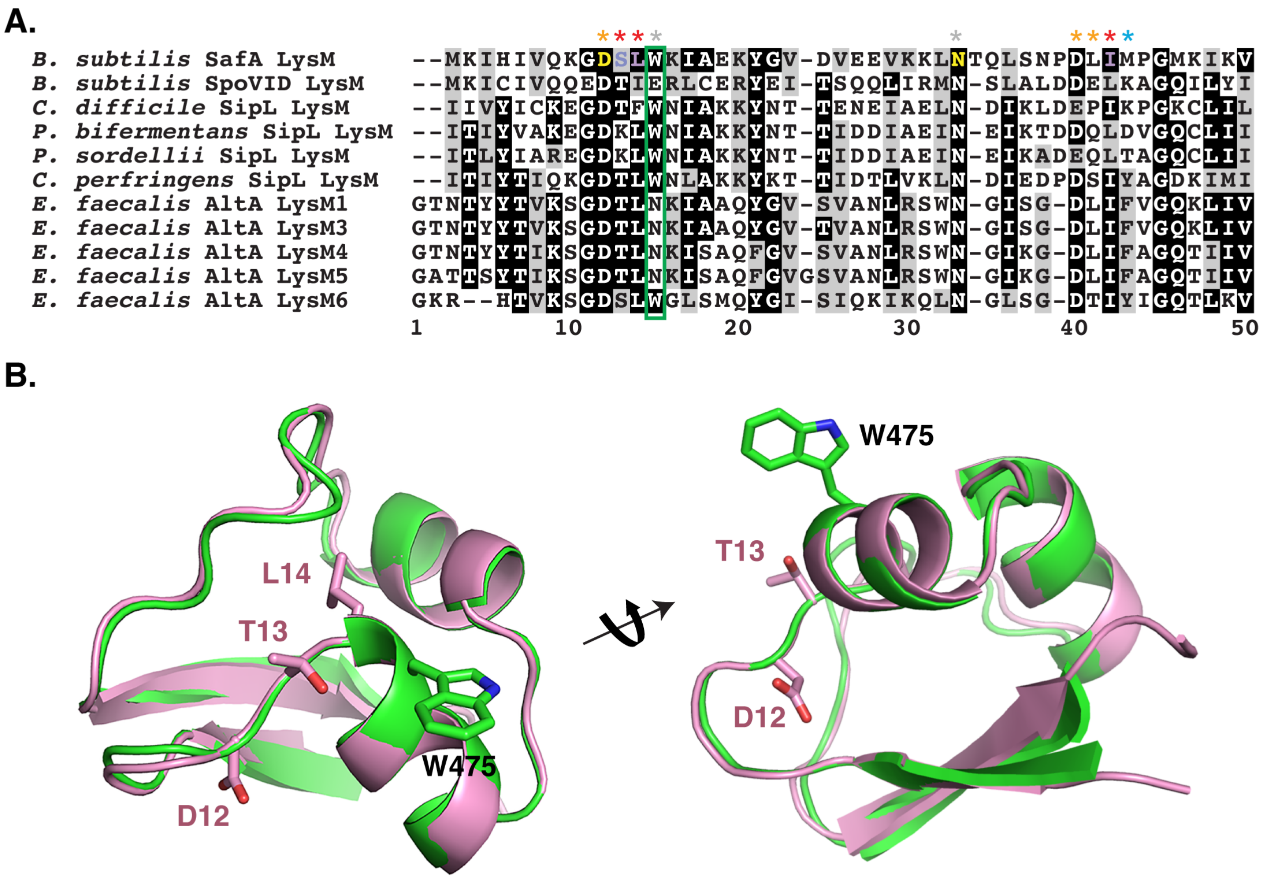

Figure S5. Comparison of LysM domains. (A) Alignment of LysM domains. The *B. subtilis* LysM domains are from SafA (NP_390662) and SpoVID (NP_390689). The clostridial LysM domains are from *C. difficile* SipL (CAJ70473) homologs from *P. bifermentans* (EQK49575)*, P. sordellii* (EPZ54296), and *C. perfringens* (YP_696893). The *Enterococcus faecalis* LysM domains are from AltA (AAA67325). Conserved residues are boxed in black with white text, and similar residues are boxed in grey with black text. SafA LysM domain residues that have been functionally characterized (4) are colored: the aspartate and asparagine residues in yellow are important for binding to SpoVID; the leucine and isoleucine residues shown in mauve are important for binding cortex; and the serine residue shown in periwinkle enhances binding to the cortex (4). The asterisks shown above the sequence indicate residues that facilitate binding of *E. faecalis* AltA LysM domains to N-acetylglucosamine (NAG) moieties (5). Red asterisks indicate

residues whose mutation to alanine resulted in severe decreases in NAG binding ~99%; orange asterisks highlight residues whose mutation to alanine decreased NAG binding by ~90%; blue asterisks highlight residues whose mutation slightly decreased NAG binding by (~25-40%); and grey asterisks highlight residues whose mutation minimally decreased NAG binding (~75-90%). The Trp475 residue of *C. difficile* LysM shown to be important for binding SpoIVA is boxed in green. (B) Superimposition of AltA LysM domain (PDB code: 4UZ2, pink) with homology model generated for *C. difficile* $\mathrm{SipL}_{\mathrm{LysM}}$ (green). The AltA residues shown are critical for binding NAG and directly interact with peptidoglycan in structural studies (5).

***sipL* complementation constructs**

pMTL-YN1C-*sipL*$_{\Delta lysM}$. Primer pair #2165 and 2176 were used to amplify the region spanning 318 bp upstream of *sipL* and the *sipL* gene until codon 462 after which a stop codon was introduced before the XhoI site. The PCR product was cloned into pMTL-YN1C digested with NotI and XhoI using Gibson assembly.

pMTL-YN1C-*sipL*$_{\Delta lysM}$*-mCherry*. Primer pair #2165 and 2260 was used to amplify regions spanning 318 bp upstream of *sipL* and the *sipL* gene. Primer pair #2259 and 2133 were used to amplify a codon-optimized *mCherry* gene derived from (6). The PCR products were fused together using PCR SOE (7) and the resulting PCR product was cloned into pMTL-YN1C digested with NotI and XhoI using Gibson assembly.

pMTL-YN1C-*sipL-B. subtilis lysM*. Primer pair #2165 and 2308 was used to amplify a region spanning 318 bp upstream of *sipL* and the *sipL* gene up until codon 462. Primer pair #2307 and 2175 were used to amplify a region encoding the *B. subtilis* SpoVID LysM domain. The PCR products were cloned into pMTL-YN1C digested with NotI and XhoI using Gibson assembly.

pMTL-YN1C-*sipL-P. bifermentans lysM*. Primer pair #2165 and 2302 was used to amplify a region spanning 318 bp upstream of *sipL* and the *sipL* gene up until codon 462. Primer pair #2301 and 2173 were used to amplify a region encoding the *P. bifermentans* SipL LysM domain. The PCR products were cloned into pMTL-YN1C digested with NotI and XhoI using Gibson assembly.

pMTL-YN1C-*sipL-C. perfringens lysM*. Primer pair #2165 and 2304 was used to amplify a region spanning 318 bp upstream of *sipL* and the *sipL* gene up until codon 462. Primer pair #2303 and 2174 were used to amplify a region encoding the *C. perfringens* SipL LysM domain. The PCR products were cloned into pMTL-YN1C digested with NotI and XhoI using Gibson assembly.

pMTL-YN1C-*sipL-P. sordellii lysM*. Primer pair #2165 and 2304 was used to amplify a region spanning 318 bp upstream of *sipL* and the *sipL* gene up until codon 462. Primer pair #2303 and 2174 were used to amplify a region encoding the *P. sordellii* SipL Lys domain. The PCR products were cloned into pMTL-YN1C digested with NotI and XhoI using Gibson assembly.

pMTL-YN1C-*sipL-3xFLAG*. Primer pair #2165 and 2300 was used to amplify a region spanning 318 bp upstream of *sipL* and the *sipL* gene lacking the stop codon. This PCR product was assembled with g-block #1, which encodes three successive codon-optimized FLAG epitope tags, and cloned into pMTL-YN1C digested with NotI and XhoI via Gibson.

pMTL-YN1C-*sipL*$_{\Delta lysM}$*-mCherry*. Primer pair #2165 and 2260 was used to amplify a region spanning 318 bp upstream of *sipL* and the *sipL* gene up until codon 462. Primer pair #2259 and 2133 were used to amplify a codon-optimized *mCherry* gene derived from (6). The PCR products were cloned into pMTL-YN1C digested with NotI and XhoI using Gibson assembly.

pMTL-YN1C-*sipL*$_{\Delta lysM}$*-3xFLAG*. Primer pair #2165 and 2402 was used to amplify a region spanning 318 bp upstream of *sipL* and the *sipL* gene lacking the stop codon. This PCR product was assembled with g-block #13, which encodes three successive codon-optimized FLAG epitope tags, into pMTL-YN1C digested with NotI and XhoI via Gibson.

pMTL-YN1C-*sipL-lysM* (aa 463-482) – Region A. Primer pair #2165 and 2300 was used to amplify a region spanning 318 bp upstream of *sipL* and the *sipL* gene until codon 462. This PCR product was assembled with g-block #2 and cloned into pMTL-YN1C digested with NotI and XhoI via Gibson.

pMTL-YN1C-*sipL-lysM* (aa 463-476) – Region B. Primer pair #2165 and 2300 was used to amplify a region spanning 318 bp upstream of *sipL* and the *sipL* gene until codon 462. This PCR product was assembled with g-block #3 and cloned into pMTL-YN1C digested with NotI and XhoI via Gibson.

pMTL-YN1C-*sipL-lysM* (aa 475-476) – Region C. Primer pair #2165 and 2300 was used to amplify a region spanning 318 bp upstream of *sipL* and the *sipL* gene until codon 462. This PCR product was assembled with g-block #4 and cloned into pMTL-YN1C digested with NotI and XhoI via Gibson.

pMTL-YN1C-*sipL-lysM* (aa 478-480) – Region D. Primer pair #2165 and 2300 was used to amplify a region spanning 318 bp upstream of *sipL* and the *sipL* gene until codon 462. This PCR product was assembled with g-block #5 and cloned into pMTL-YN1C digested with NotI and XhoI via Gibson.

pMTL-YN1C-*sipL-lysM* (aa 478-480) – Region E. Primer pair #2165 and 2300 was used to amplify a region spanning 318 bp upstream of *sipL* and the *sipL* gene until codon 462. This PCR product was assembled with g-block #10 and cloned into pMTL-YN1C digested with NotI and XhoI via Gibson.

pMTL-YN1C-*sipL*$_{\mathbf{I463E}}$. Primer pair #2165 and 2367 and primer pair #2366 and 2166 were used to amplify regions spanning 318 bp upstream of *sipL* and the *sipL* gene including the stop codon. Primers 2366 and 2367 encode the I463A mutation. The PCR products were cloned into pMTL-YN1C digested with NotI and XhoI using Gibson assembly.

pMTL-YN1C-*sipL*$_{\mathbf{I463R}}$. Primer pair #2165 and 2369 and primer pair #2368 and 2166 were used to amplify regions spanning 318 bp upstream of *sipL* and the *sipL* gene including the stop codon. Primers 2368 and 2369 encode the I463R mutation. The PCR products were cloned into pMTL-YN1C digested with NotI and XhoI using Gibson assembly.

pMTL-YN1C-*sipL*$_{\mathbf{I463M}}$. Primer pair #2165 and 2371 and primer pair #2370 and 2166 were used to amplify regions spanning 318 bp upstream of *sipL* and the *sipL* gene including the stop codon. Primers 2370 and 2371 encode the I463R mutation. The PCR products were cloned into pMTL-YN1C digested with NotI and XhoI using Gibson assembly.

pMTL-YN1C-*sipL*$_{\mathbf{W475E}}$. Primer pair #2165 and 2300 was used to amplify a region spanning 318 bp upstream of *sipL* and the *sipL* gene until codon 462. This PCR product was assembled with g-block #8 and cloned into pMTL-YN1C digested with NotI and XhoI via Gibson.

pMTL-YN1C-*sipL*$_{\mathbf{K479E}}$. Primer pair #2165 and 2300 was used to amplify a region spanning 318 bp upstream of *sipL* and the *sipL* gene until codon 462. This PCR product was assembled with g-block #9 and cloned into pMTL-YN1C digested with NotI and XhoI via Gibson.

pMTL-YN1C-*sipL*$_{\mathbf{\Delta I463}}$. Primer pair #2165 and 2400 was used to amplify a region spanning 318 bp upstream of *sipL* and the *sipL* gene until codon 462. This PCR product was assembled with g-block #14 and cloned into pMTL-YN1C digested with NotI and XhoI via Gibson.

pMTL-YN1C-*sipL*$_{\mathbf{G471E}}$. Primer pair #2165 and 2406 was used to amplify the region spanning 318 bp upstream of *sipL* to around the G471E codon. Primer pair #2405 and 695 was used to amplify the downstream region including 102 bp downstream of the end of the *sipL* gene. Primers 2406 and 2407 encode the G471E mutation. The flanking primers #2165 and 2166 were used in a PCR SOE reaction to generate the product cloned into pMTL-YN1C digested with NotI and XhoI using Gibson assembly.

pMTL-YN1C-*sipL*$_{\mathbf{E485S}}$. Primer pair #2165 and 2417 was used to amplify the region spanning 318 bp upstream of *sipL* to around the E485 codon. Primer pair #2416 and 695 was used to amplify the downstream region including 102 bp downstream of the end of the *sipL* gene. Primers 2416 and 2417 encode the E485S mutation. The flanking primers #2165 and 2166 were used in a PCR SOE reaction to generate the product cloned into pMTL-YN1C digested with NotI and XhoI using Gibson assembly.

pMTL-YN1C-*sipL*$_{\mathbf{Y466C}}$. Primer pair #2165 and 2404 was used to amplify the region spanning 318 bp upstream of *sipL* to around the Y466C codon. Primer pair #2403 and 695 was used to amplify the downstream region including 102 bp downstream of the end of the *sipL* gene. Primers 2403 and 2404 encode the Y466C mutation. The flanking primers #2165 and 2166 were used in a PCR SOE reaction to generate the product cloned into pMTL-YN1C digested with NotI and XhoI using Gibson assembly.

pMTL-YN1C-*sipL*$_{\mathbf{N482E}}$. Primer pair #2165 and 2425 was used to amplify the region spanning 318 bp upstream of *sipL* to around the N482E codon. Primer pair #2424 and 695 was used to amplify the downstream region including 102 bp downstream of the end of the *sipL* gene. Primers 2424 and 2425 encode the N482E mutation. The flanking primers #2165 and 2166 were used in a PCR SOE reaction to generate the product cloned into pMTL-YN1C digested with NotI and XhoI using Gibson assembly.

pMTL-YN1C-*sipL*$_{\mathbf{T483I}}$. Primer pair #2165 and 2419 was used to amplify the region spanning 318 bp upstream of *sipL* to around the T483I codon. Primer pair #2418 and 695 was used to amplify the downstream region including 102 bp downstream of the end of the *sipL* gene. Primers 2418 and 2419 encode the T483I mutation. The flanking primers #2165 and 2166 were used in a PCR SOE reaction to generate the product cloned into pMTL-YN1C digested with NotI and XhoI using Gibson assembly.

pMTL-YN1C-*sipL*$_{W475A}$. Primer pair #2165 and 2300 was used to amplify a region spanning 318 bp upstream of *sipL* and the *sipL* gene until codon 462. This PCR product was assembled with g-block #16 and cloned into pMTL-YN1C digested with NotI and XhoI via Gibson.

pMTL-YN1C-*sipL*$_{W475D}$. Primer pair #2165 and 2300 was used to amplify a region spanning 318 bp upstream of *sipL* and the *sipL* gene until codon 462. This PCR product was assembled with g-block #17 and cloned into pMTL-YN1C digested with NotI and XhoI via Gibson.

pMTL-YN1C-*sipL*$_{W475F}$. Primer pair #2165 and 2620 was used to amplify the region spanning 318 bp upstream of *sipL* to around the W475F codon. Primer pair #2619 and 695 was used to amplify the downstream region including 102 bp downstream of the end of the *sipL* gene. Primers 2619 and 2620 encode the W475F mutation. The flanking primers #2165 and 2166 were used in a PCR SOE reaction to generate the product cloned into pMTL-YN1C digested with NotI and XhoI using Gibson assembly.

pMTL-YN1C-*sipL*$_{W475E}$-*3XFLAG*. Primer pair #2165 and 2300 was used to amplify a region spanning 318 bp upstream of *sipL* and the *sipL* gene until codon 462. This PCR product was assembled with g-block #23 and cloned into pMTL-YN1C digested with NotI and XhoI via Gibson.

pMTL-YN1C-*sipL*$_{\Delta I463}$-*3XFLAG*. Primer pair #2165 and 2300 was used to amplify a region spanning 318 bp upstream of *sipL* and the *sipL* gene until codon 462. This PCR product was assembled with g-block #25 and cloned into pMTL-YN1C digested with NotI and XhoI via Gibson.

pMTL-YN1C-*sipL*$_{I463R}$-*3XFLAG*. Primer pair #2165 and 2300 was used to amplify a region spanning 318 bp upstream of *sipL* and the *sipL* gene until codon 462. This PCR product was assembled with g-block #24 and cloned into pMTL-YN1C digested with NotI and XhoI via Gibson.

Supplementary Table S1. Strains used in this study

Strain #	Strain name	Relevant genotype or features	Source/reference
C. difficile* strains – 630Δ*erm			
805	630Δ*erm*Δ*pyrE* Δ*spoIVA*	630Δ*erm*Δ*pyrE* with *spoIVA* (*CD2629*) deleted	(3)
846	630Δ*erm*-p	*erm*-sensitive derivate of 630 with *pyrE* restored	(8)
849	630Δ*erm* Δ*spo0A*-p	630Δ*erm*Δ*spo0A* with *pyrE* restored	(8)
1005	630Δ*erm*Δ*pyrE* Δ*sipL*	630Δ*erm*Δ*pyrE* with *sipL* deleted	(9)
1010	630Δ*erm* Δ*sipL*-p	630Δ*erm*Δ*pyrE* with *sipL* deleted	(9)
1013	630Δ*erm* Δ*sipL*/*sipL*	630Δ*erm*Δ*sipL* with *sipL* in the *pyrE* locus	(9)
1117	630Δ*erm* Δ*sipL*/*sipL*$_{\Delta lysM}$	630Δ*erm*Δ*sipL* with *sipL*$_{\Delta lysM}$ in the *pyrE* locus	This study
1144	630Δ*erm*/*mCherry-IVA*	630Δ*erm* with *mCherry-IVA* in the *pyrE* locus	**(3)**
1158	630Δ*erm* Δ*sipL*/*sipL-mCherry*	630Δ*erm* Δ*sipL* with *sipL-mCherry* in the *pyrE* locus	(9)
1211	630Δ*erm* Δ*sipL*/*mCherry-IVA*	630Δ*erm* Δ*sipL* with *mCherry-IVA* in the *pyrE* locus	This study
1244	630Δ*erm* Δ*sipL*/*sipL*$_{\Delta lysM}$*-mCherry*	630Δ*erm* Δ*sipL* with *sipL*$_{\Delta lysM}$*-mCherry* in the *pyrE* locus	This study
1365	630Δ*erm* Δ*sipL*/*sipL-lysM sub*	630Δ*erm* Δ*sipL*/*sipL-lysM B. subtilis* in the *pyrE* locus	This study
1368	630Δ*erm* Δ*sipL*/*sipL-lysM bif*	630Δ*erm* Δ*sipL*/*sipL-lysM P. bifermentans* in the *pyrE* locus	This study
1371	630Δ*erm* Δ*sipL*/*sipL-lysM perf*	630Δ*erm* Δ*sipL*/*sipL-lysM C. perfringens* in the *pyrE* locus	This study
1374	630Δ*erm* Δ*sipL*/*sipL-lysM sor*	630Δ*erm* Δ*sipL*/*sipL-lysM P. sordellii* in the *pyrE* locus	This study
1852	630Δ*erm* Δ*sipL*/*sipL-3XFLAG*	630Δ*erm* Δ*sipL* with *sipL-3XFLAG* in the *pyrE* locus	This study
1405	630Δ*erm*Δ*pyrE* Δ*spoIVA* Δ*sipL*	630Δ*erm*Δ*pyrE*Δ*spoIVA* with *sipL* deleted	This study
1410	630Δ*erm* Δ*IVA*Δ*sipL*/*sipL-mCherry*	630Δ*erm* Δ*spoIVA*Δ*sipL* with *sipL-mCherry* in the *pyrE* locus	This study
1421	630Δ*erm* Δ*sipL*/*sipL*(463-482)	630Δ*erm* Δ*sipL* with *sipL*(463-482) Region A in the *pyrE* locus	This study
1424	630Δ*erm* Δ*sipL*/*sipL*(463-476)	630Δ*erm* Δ*sipL* with *sipL*(463-476) Region B in the *pyrE* locus	This study
1427	630Δ*erm* Δ*sipL*/*sipL*(475-476)	630Δ*erm* Δ*sipL* with *sipL*(475-476) Region C in the *pyrE* locus	This study
1430	630Δ*erm* Δ*sipL*/*sipL*(478-480)	630Δ*erm* Δ*sipL* with *sipL*(463-476) Region D in the *pyrE* locus	This study
1453	630Δ*erm* Δ*sipL*/*sipL*$_{I463E}$	630Δ*erm*Δ*sipL* with *sipL*$_{I463E}$ in the *pyrE* locus	This study
1456	630Δ*erm* Δ*sipL*/*sipL*$_{I463R}$	630Δ*erm*Δ*sipL* with *sipL*$_{I463R}$ in the *pyrE* locus	This study
1459	630Δ*erm* Δ*sipL*/*sipL*$_{I463M}$	630Δ*erm*Δ*sipL* with *sipL*$_{I463M}$ in the *pyrE* locus	This study
1500	630Δ*erm* Δ*sipL*/*sipL*$_{W475E}$	630Δ*erm*Δ*sipL* with *sipL*$_{W475E}$ in the *pyrE* locus	This study
1503	630Δ*erm* Δ*sipL*/*sipL*$_{K479E}$	630Δ*erm*Δ*sipL* with *sipL*$_{K479E}$ in the *pyrE* locus	This study
1506	630Δ*erm* Δ*sipL*/*sipL*(483-516)	630Δ*erm* Δ*sipL* with *sipL*(486-516) Region E in the *pyrE* locus	This study
1536	630Δ*erm* Δ*sipL*/*sipL*$_{\Delta lysM}$*-3XFLAG*	630Δ*erm* Δ*sipL* with *sipL*$_{\Delta lysM}$ *-3XFLAG* in the *pyrE* locus	This study
1560	630Δ*erm* Δ*sipL*/*sipL*$_{\Delta I463}$	630Δ*erm*Δ*sipL* with *sipL*$_{\Delta I463}$ in the *pyrE* locus	This study
1575	630Δ*erm* Δ*sipL*/*sipL*$_{G471E}$	630Δ*erm*Δ*sipL* with *sipL*$_{G471E}$ in the *pyrE* locus	This study
1578	630Δ*erm* Δ*sipL*/*sipL*$_{E485S}$	630Δ*erm*Δ*sipL* with *sipL*$_{E485S}$ in the *pyrE* locus	This study
1651	630Δ*erm* Δ*sipL*/*sipL*$_{Y466C}$	630Δ*erm*Δ*sipL* with *sipL*$_{Y466C}$ in the *pyrE* locus	This study
1654	630Δ*erm* Δ*sipL*/*sipL*$_{N482E}$	630Δ*erm*Δ*sipL* with *sipL*$_{N482E}$ in the *pyrE* locus	This study
1657	630Δ*erm* Δ*sipL*/*sipL*$_{T483I}$	630Δ*erm*Δ*sipL* with *sipL*$_{T483I}$ in the *pyrE* locus	This study
1708	630Δ*erm* Δ*sipL*/*sipL*$_{W475A}$	630Δ*erm*Δ*sipL* with *sipL*$_{W475A}$ in the *pyrE* locus	This study
1711	630Δ*erm* Δ*sipL*/*sipL*$_{W475D}$	630Δ*erm*Δ*sipL* with *sipL*$_{W475D}$ in the *pyrE* locus	This study
1938	630Δ*erm* Δ*sipL*/*sipL*$_{W475F}$	630Δ*erm*Δ*sipL* with *sipL*$_{W475F}$ in the *pyrE* locus	This study
2032	630Δ*erm* Δ*sipL*/*sipL*$_{W475E}$*-3XFLAG*	630Δ*erm* Δ*sipL* with *sipL*$_{W475E}$*-3XFLAG* in the *pyrE* locus	This study
2035	630Δ*erm* Δ*sipL*/*sipL*$_{I463R}$*-3XFLAG*	630Δ*erm* Δ*sipL* with *sipL*$_{I463R}$ *-3XFLAG* in the *pyrE* locus	This study
2038	630Δ*erm* Δ*sipL*/*sipL*$_{\Delta I463}$*-3XFLAG*	630Δ*erm* Δ*sipL* with *sipL*$_{\Delta I463}$*-3XFLAG* in the *pyrE* locus	This study

***E. coli* strains**

Strain #	Strain name	Relevant genotype or features	Source
41	DH5α	F– Φ80*lacZ*ΔM15 Δ(*lacZYA-argF*) U169 *recA1 endA1 hsdR17* (rK^-, mK^+) *phoA supE44* λ– *thi-1 gyrA96 relA1*	D. Cameron

531	HB101/pRK24	F- *mcrB mrr hsdS20*(rB^{-}mB^{-}) *recA13 leuB6 ara-13 proA2 lavYI galK2 xyl-6 mtl-1 rpsL20* carrying pRK24	C. Ellermeier
764	pET21a-SipL$_{\Delta \mathrm{LysM}}$	pET21a-SipL$_{\Delta \mathrm{LysM}}$ in BL21(DE3)	(10)
1539	pMTL-YN3	pMTL-YN3 in DH5α	(11)
1704	pMTL-YN3 Δ*sipL*	pMTL-YN3 Δ*sipL* in HB101	(9)
1754	pMTL-YN1C *sipL*$_{\Delta lysM}$	pMTL-YN1C *sipL*$_{\Delta lysM}$ in HB101	This study
1768	pMTL-YN1C *mCherry-IVA*	pMTL-YN1C *mCherry-spoIVA*	(3)
1777	pMTL-YN1C *sipL-mCherry*	pMTL-YN1C *sipL-mCherry* in HB101	(9)
1815	pMTL-YN1C *sipL*$_{\Delta lysM}$*-mCherry*	pMTL-YN1C *sipL*$_{\Delta lysM}$*-mCherry* in HB101	This study
1847	pMTL-YN1C *sipL-lysM sub*	pMTL-YN1C *sipL-lysM B. subtilis* in HB101	This study
1849	pMTL-YN1C *sipL-lysM bif*	pMTL-YN1C *sipL-lysM P. bifermentans* in HB101	This study
1851	pMTL-YN1C *sipL-lysM perf*	pMTL-YN1C *sipL-lysM C. perfringens* in HB101	This study
1858	pMTL-YN1C *sipL-lysM sor*	pMTL-YN1C *sipL-lysM P. sordellii* in HB101	This study
1859	pMTL-YN1C *sipL-3xFLAG*	pMTL-YN1C *sipL-3xFLAG* in HB101	This study
1879	pMTL-YN1C *sipL*(463-482)	pMTL-YN1C *sipL*(463-482) Region A in HB101	This study
1881	pMTL-YN1C *sipL*(463-476)	pMTL-YN1C *sipL*(463-476) Region B in HB101	This study
1883	pMTL-YN1C *sipL*(475-476)	pMTL-YN1C *sipL*(475-476) Region C in HB101	This study
1885	pMTL-YN1C *sipL*(478-480)	pMTL-YN1C *sipL*(478-480) Region D in HB101	This study
1894	pMTL-YN1C *sipL*$_{\mathrm{I463E}}$	pMTL-YN1C *sipL*$_{\mathrm{I463E}}$ in HB101	This study
1896	pMTL-YN1C *sipL*$_{\mathrm{I463R}}$	pMTL-YN1C *sipL*$_{\mathrm{I463R}}$ in HB101	This study
1898	pMTL-YN1C *sipL*$_{\mathrm{I463M}}$	pMTL-YN1C *sipL*$_{\mathrm{I463M}}$ in HB101	This study
1911	pMTL-YN1C *sipL*$_{\mathrm{W475E}}$	pMTL-YN1C *sipL*$_{\mathrm{W475E}}$ in HB101	This study
1913	pMTL-YN1C *sipL*$_{\mathrm{K479E}}$	pMTL-YN1C *sipL*$_{\mathrm{K479E}}$ in HB101	This study
1915	pMTL-YN1C *sipL*(483-516)	pMTL-YN1C *sipL*(483-516) Region E in HB101	This study
1926	pMTL-YN1C *sipL-*Δ_{lysM}*3xFLAG*	pMTL-YN1C *sipL-*$_{\Delta lysM}$*3xFLAG* in HB101	This study
1928	pMTL-YN1C *sipL*$_{\Delta \mathrm{I463}}$	pMTL-YN1C *sipL*$_{\Delta \mathrm{I463}}$ in HB101	This study
1938	pMTL-YN1C *sipL*$_{\mathrm{G471E}}$	pMTL-YN1C *sipL*$_{\mathrm{G471E}}$ in HB101	This study
1940	pMTL-YN1C *sipL*$_{\mathrm{E485S}}$	pMTL-YN1C *sipL*$_{\mathrm{E485S}}$ in HB101	This study
1981	pMTL-YN1C *sipL*$_{\mathrm{W475A}}$	pMTL-YN1C *sipL*$_{\mathrm{W475A}}$ in HB101	This study
1982	pMTL-YN1C *sipL*$_{\mathrm{W475D}}$	pMTL-YN1C *sipL*$_{\mathrm{W475D}}$ in HB101	This study
2083	pMTL-YN1C *sipL*$_{\mathrm{Y466C}}$	pMTL-YN1C *sipL*$_{\mathrm{Y466C}}$ in HB101	This study
2084	pMTL-YN1C *sipL*$_{\mathrm{N482E}}$	pMTL-YN1C *sipL*$_{\mathrm{N482E}}$ in HB101	This study
2085	pMTL-YN1C *sipL*$_{\mathrm{T483I}}$	pMTL-YN1C *sipL*$_{\mathrm{T483I}}$ in HB101	This study
2111	pMTL-YN1C *sipL*$_{\mathrm{W475F}}$	pMTL-YN1C *sipL*$_{\mathrm{W475F}}$ in HB101	This study
2141	pMTL-YN1C *sipL*$_{\mathrm{W475E}}$*-3xFLAG*	pMTL-YN1C *sipL*$_{\mathrm{W475E}}$*-3xFLAG* in HB101	This study
2143	pMTL-YN1C *sipL*$_{\mathrm{I463R}}$*-3xFLAG*	pMTL-YN1C *sipL*$_{\mathrm{I463R}}$*-3xFLAG* in HB101	This study
2145	pMTL-YN1C *sipL*$_{\Delta \mathrm{I463}}$*-3xFLAG*	pMTL-YN1C *sipL*$_{\Delta \mathrm{I463}}$*-3xFLAG* in HB101	This study

Supplementary Table S2. Primers used in the study

Number	Primer name	
695	3’ XhoI *sipL* 102 bp downstream	AACGCTCGAGTAAAGAATAATGCATCACTAAC
2133	3' XhoI mCherry Gibson	gccaagcttgcatgtctgcaggcctcgagTTATTTATATAATTCATCCATACCTCCTGTTG
2165	5' NotI *sipL* Gibson	ggaattagggatgtaataagcggccgcGAGAGACATATAGGAAAGAAAATATTG
2166	3' XhoI *sipL* Gibson	gcttgcatgtctgcaggcctcgagTTAATCTACTAATACGACTTTTTTTCTAAAATAAG
2173	3' XhoI Cbif *lysM* YN1C Gibson	gcttgcatgtctgcaggcctcgagTTATACTTCTACTGGAGTTTTTTTC
2174	3' XhoI Cperf *lysM* YN1C Gibson	gcttgcatgtctgcaggcctcgagTTATAAAACTGCTCTTCCTGGAATC
2175	3' XhoI Bsub *lysM* YN1C Gibson	gcttgcatgtctgcaggcctcgagTTACGCATGGCTATTTTTATATTGAGG
2176	3' XhoI *sipLΔlysM* Gibson	gcttgcatgtctgcaggcctcgagTTAGCTAGGAGCTTTAGATAAATCATATACACC
2259	5' *sipLΔlysM*-mCherry SOE	GTGTATATGATTTATCTAAAGCTCCTAGCGCAATGGTATCTAAAGGAGAAGAAG
2260	3' *sipLΔlysM*-mCherry rev eos	CTTCTTCTCCTTTAGATACCATTGCGCTAGGAGCTTTAGATAAATCATATACAC
2300	3' *sipL* LysM portion rev eos	CGATTATGCTAGGAGCTTTAGATAAATCATATACACCTTGGTCTTCTCCCTTG
2301	5' Cdif *sipL* Cbif lysM SOE	GATTTATCTAAAGCTCCTAGCATAACGATATATGTTGCTAAAG
2302	3' Cdif *sipL* Cbif lysM rev eos	CTTTAGCAACATATATCGTTATGCTAGGAGCTTTAGATAAATC
2303	5' Cdif *sipL* Cperf lysM SOE	GATTTATCTAAAGCTCCTAGCATTACTATATATACAATTCAAAAAGG
2304	3' Cdif *sipL* Cperf lysM rev eos	CCTTTTTGAATTGTATATATAGTAATGCTAGGAGCTTTAGATAAATC
2305	5' Cdif *sipL* Csor lysM SOE	GATTTATCTAAAGCTCCTAGCATAACTTTGTATATAGCTAGAG
2306	3' Cdif *sipL* Csor lysM rev eos	CTCTAGCTATATACAAAGTTATGCTAGGAGCTTTAGATAAATC
2307	5' Cdif *sipL* Bsub lysM SOE	GATTTATCTAAAGCTCCTAGCATGAAAATTTGTATTGTGCAGC
2308	3' Cdif *sipL* Bsub lysM rev eos	GCTGCACAATACAAATTTTCATGCTAGGAGCTTTAGATAAATC
2323	3' XhoI Csor *lysM* YN1C Gibson	gcttgcatgtctgcaggcctcgagTTAAGCTTCTATAAGCGTTTTTTTC
2366	5' YN1C Cdiff *sipL* I463E SOE QC	GATTTATCTAAAGCTCCTAGCGAAATCGTATATATATGCAAAGAAGGAG
2367	3' YN1C Cdiff *sipL* I463E SOE QC rev	CTCCTTCTTTGCATATATATACGATTTCGCTAGGAGCTTTAGATAAATC
2368	5' YN1C Cdiff *sipL* I463R SOE QC	GATTTATCTAAAGCTCCTAGCAGAATCGTATATATATGCAAAGAAGGAG
2369	3' YN1C Cdiff *sipL* I463R SOE QC rev	CTCCTTCTTTGCATATATATACGATTCTGCTAGGAGCTTTAGATAAATC
2370	5' YN1C Cdiff *sipL* I463M SOE QC	GATTTATCTAAAGCTCCTAGCATGATCGTATATATATGCAAAGAAGGAG
2371	3' YN1C Cdiff *sipL* I463M SOE QC rev	CTCCTTCTTTGCATATATATACGATCATGCTAGGAGCTTTAGATAAATC
2400	3' sipL ΔI463 rev SOE	ATACGATGCTAGGAGCTTTAGATAAATCATATACACCTTGGTCTTCTCCCTTG
2402	3' *sipLΔlysM*-3xFLAG rev eos	GAAAATTTATGGAAAAGTTGGTTATACCTAATATAGACAATGAATTTAAAAAGGATAG
2402	3' *sipLΔlysM* -3xFLAG rev eos	GCTGTATCAGTTAGATTATCCATTTCTATATCATGTTCAAATGGAATTTCCTC
2403	5' *sipL* Y466C SOE	AGCATAATCGTATGTATATGCAAAGAAGGAGATACTTTCTGG
2404	3' *sipL* Y466C rev eos	CTCCTTCTTTGCATATACATACGATTATGCTAGGAGCTTTAG
2405	5' *sipL* G471E SOE	CGTATATATATGCAAAGAAGAAGATACTTTCTGGAATATAGC
2406	3' *sipL* G471E rev eos	CAGAAAGTATCTTCTTCTTTGCATATATATACGATTATGCTAGG

2416	5' *sipL* E485S SOE	CAATACAACAAGTAATGAAATTGCAGAACTTAATGATATAAAAC
2417	3' *sipL* E485S rev eos	CATTAAGTTCTGCAATTTCATTACTTGTTGTATTGTATTTTTTTG
2418	5' *sipL* E487Q SOE	CAGAAAATCAAATTGCAGAACTTAATGATATAAAACTTGATGAAC
2419	3' *sipL* E487Q rev eos	GTTTTATATCATTAAGTTCTGCAATTTGATTTTCTGTTGTATTG
2424	5' *sipL* T483I SOE	GCAAAAAAATACAATATAACAGAAAATGAAATTGCAGAACTTAATG
2425	3' *sipL* T483I rev eos	CTGCAATTTCATTTTCTGTTATATTGTATTTTTTTGCTATATTCCAG
2619	5' *sipL* W475F SOE	GCAAAGAAGGAGATACTTTCTTTAATATAGCAAAAAAATACAATACAACAG
2620	3' *sipL* W475F rev eos	CTGTTGTATTGTATTTTTTTGCTATATTAAAGAAAGTATCTCCTTCTTTGC

Restriction sites are underlined.

Lower case letters indicate sequences derived from the pMTL-YN1C vector for Gibson assembly.

Supplementary Table S3. gBlocks.

gblock #	Name	Sequence
1	LysM-3xFLAG	CAAGGGAGAAGACCAAGGTGTATATGATTTATCTAAAGCTCCTAGCATAATCGTATATATATGCAAAGAAGGAGATACTTTCTGGAATATAGCAAAAAAATACAATACAA<br>CAGAAAATGAAATTGCAGAACTTAATGATATAAAACTTGATGAACCAATTAAGCCTGGGAAGTGTCTTATTTTAGAAAAAAAAGTCGTATTAGTAGATGATTATAAAGAT<br>GATGATGATAAAGACTATAAAGATGACGATGATAAGGATTATAAGGATGATGATGACAAATAActcgaggcctgcagacatgcaagcttggc
2	LysM aa 463-482 (Region A)	CAAGGGAGAAGACCAAGGTGTATATGATTTATCTAAAGCTCCTAGCATGAAAATTTGTATTGTGCAGCAGGAAGATACGATCGAGCGTTTATGCGAACGGTATGAAACAA<br>CAGAAAATGAAATTGCAGAACTTAATGATATAAAACTTGATGAACCAATTAAGCCTGGGAAGTGTCTTATTTTAGAAAAAAAAGTCGTATTAGTAGATTAActcgaggcctgcaga<br>catgcaagcttggc
3	LysM aa 463-476 (Region B)	CAAGGGAGAAGACCAAGGTGTATATGATTTATCTAAAGCTCCTAGCATGAAAATTTGTATTGTGCAGCAGGAAGATACGATCGAGCGTATAGCAAAAAAATACAATACAA<br>CAGAAAATGAAATTGCAGAACTTAATGATATAAAACTTGATGAACCAATTAAGCCTGGGAAGTGTCTTATTTTAGAAAAAAAAGTCGTATTAGTAGATTAActcgaggcctgcaga<br>catgcaagcttggc
4	LysM aa 475-476 (Region C)	CAAGGGAGAAGACCAAGGTGTATATGATTTATCTAAAGCTCCTAGCATAATCGTATATATATGCAAAGAAGGAGATACTTTCGAGCGTATAGCAAAAAAATACAATACAA<br>CAGAAAATGAAATTGCAGAACTTAATGATATAAAACTTGATGAACCAATTAAGCCTGGGAAGTGTCTTATTTTAGAAAAAAAAGTCGTATTAGTAGATTAActcgaggcctgcaga<br>catgcaagcttggc
5	LysM aa 478-480 (Region D)	CAAGGGAGAAGACCAAGGTGTATATGATTTATCTAAAGCTCCTAGCATAATCGTATATATATGCAAAGAAGGAGATACTTTCTGGAATATATGCGAACGGTACAATACAA<br>CAGAAAATGAAATTGCAGAACTTAATGATATAAAACTTGATGAACCAATTAAGCCTGGGAAGTGTCTTATTTTAGAAAAAAAAGTCGTATTAGTAGATTAACTCGAGGCC<br>TGCAGACATGCAAGCTTGGC
8	W475E	CAAGGGAGAAGACCAAGGTGTATATGATTTATCTAAAGCTCCTAGCATAATCGTATATATATGCAAAGAAGGAGATACTTTCGAAAATATAGCAAAAAAATACAATACAA<br>CAGAAAATGAAATTGCAGAACTTAATGATATAAAACTTGATGAACCAATTAAGCCTGGGAAGTGTCTTATTTTAGAAAAAAAAGTCGTATTAGTAGATTAActcgaggcctgcaga<br>catgcaagcttggc
9	K479E	CAAGGGAGAAGACCAAGGTGTATATGATTTATCTAAAGCTCCTAGCATAATCGTATATATATGCAAAGAAGGAGATACTTTCTGGAATATAGCAGAAAAATACAATACAA<br>CAGAAAATGAAATTGCAGAACTTAATGATATAAAACTTGATGAACCAATTAAGCCTGGGAAGTGTCTTATTTTAGAAAAAAAAGTCGTATTAGTAGATTAActcgaggcctgcaga<br>catgcaagcttggc
10	LysM aa 483-516 (Region E)	CAAGGGAGAAGACCAAGGTGTATATGATTTATCTAAAGCTCCTAGCATAATCGTATATATATGCAAAGAAGGAGATACTTTCTGGAATATAGCAAAAAAATACAATATTA<br>CATCCCAGCAGCTGATCAGGATGAATTCTTTAGCCTTGGATGATGAATTAAAAGCAGGACAGATTCTCTATATTCCTCAATATAAAAATAGCCATGCGTAActcgaggcctgcagac<br>atgcaagcttggc
13	ΔLysM-3xFLAG	GAGGAAATTCCATTTGAACATGATATAGAAATGGATAATCTAACTGATACAGCATCTGTGTTTAATACAGCATGTATTGACAAAGTCGAGGTTGATTTGAATAGGGACCAG<br>ATAGACTTGATTATCAAGATTAAGAGATTTACTGAGGCATTAGACAAGAAAGCAGAAAATTTTATTATCAAGGGAGAAGACCAAGGTGTATATGATTTATCTAAAGCTCC<br>TAGCGATTATAAGGATGACGATGATAAAGACTATAAAGATGACGATGATAAGGATTATAAGGATGACGATGACAAATAActcgaggcctgcagacatgcaagcttggc
14	ΔI463	TATCAAGGGAGAAGACCAAGGTGTATATGATTTATCTAAAGCTCCTAGCATCGTATATATATGCAAGGAAGGAGACACTTTCTGGAACATAGCAAAGAAGTACAATACAA<br>CAGAGAATGAAATTGCAGAGCTTAATGATATAAAGCTTGATGAACCAATTAAGCCTGGGAAGTGTCTTATTTTAGAAAAAAAAGTCGTATTAGTAGATTAActcgaggcctgcaga<br>catgcaagcttggc
16	W475A	CAAGGGAGAAGACCAAGGTGTATATGATTTATCTAAAGCTCCTAGCATAATCGTATATATATGCAAAGAAGGAGATACTTTCGCAAATATAGCAAAAAAATACAATACAA<br>CAGAAAATGAAATTGCAGAACTTAATGATATAAAACTTGATGAACCAATTAAGCCTGGGAAGTGTCTTATTTTAGAAAAAAAAGTCGTATTAGTAGATTAActcgaggcctgcaga<br>catgcaagcttggcactggccgtcg

17	W475D	CAAGGGAGAAGACCAAGGTGTATATGATTTATCTAAAGCTCCTAGCATAATCGTATATATATGCAAAGAAGGAGATACTTTCGATAATATAGCAAAAAAATACAATACAA CAGAAAATGAAATTGCAGAACTTAATGATATAAAACTTGATGAACCAATTAAGCCTGGGAAGTGTCTTATTTTAGAAAAAAAAGTCGTATTAGTAGATTAActcgaggcctgcaga catgcaagcttggcactggccgtcg
23	$LysM_{W475E}$-3xFLAG	CAAGGGAGAAGACCAAGGTGTATATGATTTATCTAAAGCTCCTAGCATAATCGTATATATATGCAAAGAAGGAGATACTTTCGAAAATATAGCAAAAAAATACAATACAA CAGAAAATGAAATTGCAGAACTTAATGATATAAAACTTGATGAACCAATTAAGCCTGGGAAGTGTCTTATTTTAGAAAAAAAAGTCGTATTAGTAGATGATTATAAAGAT GATGATGATAAAGAcTATAAAGATGACGATGATAAGGATTATAAGGATGATGATGACAAATAActcgaggcctgcagacatgcaagcttggc
24	$LysM_{I463R}$-3xFLAG	CAAGGGAGAAGACCAAGGTGTATATGATTTATCTAAAGCTCCTAGCAGAATCGTATATATATGCAAAGAAGGAGATACTTTCTGGAATATAGCAAAAAAATACAATACAA CAGAAAATGAAATTGCAGAACTTAATGATATAAAACTTGATGAACCAATTAAGCCTGGGAAGTGTCTTATTTTAGAAAAAAAAGTCGTATTAGTAGATGATTATAAAGAT GATGATGATAAAGAcTATAAAGATGACGATGATAAGGATTATAAGGATGATGATGACAAATAActcgaggcctgcagacatgcaagcttggc
25	$LysM_{\Delta I463}$-3xFLAG	TATCAAGGGAGAAGACCAAGGTGTATATGATTTATCTAAAGCTCCTAGCATCGTATATATATGCAAAGAAGGAGATACTTTCTGGAATATAGCAAAAAAATACAATACAA CAGAAAATGAAATTGCAGAACTTAATGATATAAAACTTGATGAACCAATTAAGCCTGGGAAGTGTCTTATTTTAGAAAAAAAAGTCGTATTAGTAGATGATTATAAAGAT GATGATGATAAAGAcTATAAAGATGACGATGATAAGGATTATAAGGATGATGATGACAAATAActcgaggcctgcagacatgcaagcttggc

Lower case letters indicate sequences derived from the pMTL-YN1C vector for Gibson assembly.

References

1. Levin PA, Fan N, Ricca E, Driks A, Losick R, Cutting S. 1993. An unusually small gene required for sporulation by *Bacillus subtilis*. Mol Microbiol 9:761-71.
2. Fimlaid KA, Jensen O, Donnelly ML, Siegrist MS, Shen A. 2015. Regulation of *Clostridium difficile* Spore Formation by the SpoIIQ and SpoIIIA Proteins. PLoS Genet 11:e1005562.
3. Ribis JW, Ravichandran P, Putnam EE, Pishdadian K, Shen A. 2017. The Conserved Spore Coat Protein SpoVM Is Largely Dispensable in *Clostridium difficile* Spore Formation. mSphere 2.
4. Pereira FC, Nunes F, Cruz F, Fernandes C, Isidro AL, Lousa D, Soares CM, Moran CP, Jr., Henriques AO, Serrano M. 2018. A LysM domain intervenes in sequential protein-protein and protein-peptidoglycan interactions important for spore coat assembly in *Bacillus subtilis*. J Bacteriol doi:10.1128/JB.00642-18.
5. Mesnage S, Dellarole M, Baxter NJ, Rouget JB, Dimitrov JD, Wang N, Fujimoto Y, Hounslow AM, Lacroix-Desmazes S, Fukase K, Foster SJ, Williamson MP. 2014. Molecular basis for bacterial peptidoglycan recognition by LysM domains. Nat Commun 5:4269.
6. Ransom EM, Ellermeier CD, Weiss DS. 2015. Use of mCherry Red fluorescent protein for studies of protein localization and gene expression in *Clostridium difficile*. Appl Environ Microbiol 81:1652-60.
7. Horton R, Hunt H, Ho S, Pullen J, Pease L. 1989. Engineering hybrid genes without the use of restriction enzymes: gene splicing by overlap extension. Gene 77:61-68.
8. Donnelly ML, Li W, Li YQ, Hinkel L, Setlow P, Shen A. 2017. A *Clostridium difficile*-Specific, Gel-Forming Protein Required for Optimal Spore Germination. MBio 8.
9. Ribis JW, Fimlaid KA, Shen A. 2018. Differential requirements for conserved peptidoglycan remodeling enzymes during *Clostridioides difficile* spore formation. Mol Microbiol 110:370-389.
10. Putnam EE, Nock AM, Lawley TD, Shen A. 2013. SpoIVA and SipL are *Clostridium difficile* spore morphogenetic proteins. J Bacteriol 195:1214-25.
11. Ng YK, Ehsaan M, Philip S, Collery MM, Janoir C, Collignon A, Cartman ST, Minton NP. 2013. Expanding the repertoire of gene tools for precise manipulation of the *Clostridium difficile* genome: allelic exchange using *pyrE* alleles. PLoS One 8:e56051.
